# Round the Hospitals

**Published:** 1918-07-27

**Authors:** 


### ROUND THE HOSPITALS.

The controversy arising out of the efforts to secure by Act of Parliament the registration of trained nurses in this country has endured for thirty years. Its advanced supporters have become so obsessed by their efforts and connection with it that registration has become a sort of fetish to them, and they have persuaded themselves and want to persuade others that an Act of Parliament granting registration will secure British nurses practically everything they can desire. "Ierne" this week shows the absurdity of these claims, and points out that registration will not only not do what the fetish party claims, but in practice must result in very little. If an Act of Parliament granted to every trained nurse who attained a requisite standard the title of *nursing sister* as her exclusive right, that

**ROUND THE HOSPITALS—(*continued*).**

would achieve substantial service for fully trained nurses for reasons mentioned later. Thirty years is a long time for a controversy to continuously proceed. It is not surprising to find that the correspondence from the *Times* which we have already published—a further portion of it will be found on page 375—demonstrates to those who have the actual facts and documents before them, if we treat all who have taken part in it as straightforwardly honest, that one of them at any rate who claims, we understand, that he never keeps a letter or files a document, must have forgotten material facts which puts him out of court.

The enormous development and many-sidedness of hospital treatment in the present day renders it impossible for any woman, however able, to cover the whole of it and become master of her duties as a trained nurse in less than three years, throughout which to make this result possible her whole time must be spent in practical work in a modern, up-to-date hospital of the best. Unity, as "Ierne" properly insists, is essential to the progressive development of the nursing profession, and to the adequate success of the working nurse everywhere. Every matron, sister, and nurse who loves her profession and has a due regard to her own interests must therefore join the College of Nursing, and make it her business to learn to keep busy and keep busy learning, by co-operation with her sisters, to recruit twenty, thirty, or, better still, forty thousand fully qualified nurses to become members, and put their names on the College register with all possible dispatch. Then, and quickly, the trained nurse will be mistress in her own household, the sweating of nurses will cease to be possible anywhere in the British Islands, and the nurse will at length be paid adequately. Then the sister, who, being a good and capable woman, is the backbone of our hospital system to-day, will no longer have to bear the bulk of responsibility for results, while she receives less than half the pay her office should entitle her to receive.

The new forms of prayer put forth by the authority of the Archbishops of Canterbury and York for use on the Intercession Day, August 4, are again very disappointing from the medical and nursing point of view. The fatal vagueness of the former suffrage which set the nation praying on behalf of the sick and wounded merely that God would "cheer" them still lingers in the faint-hearted "if it be Thy will give them health again." Do the Archbishops hesitate to believe it is God's will that these men should be healed? When the surgeons concentrate untiringly on seemingly hopeless cases, when the nurses spend themselves to the last ounce of reserve strength to cherish the faint spark of life, are they to be denied the consolation of believing that their labours are always and entirely in fulfilment of God's will that the lives He gave may be preserved? The proviso "if it be Thy will," suggested by the Apostle as appropriate in making personal plans, strikes at the whole root of the healing art when used in this connection. It takes man back to primeval notions of God as the dispenser of plagues and "sundry kinds of death." This proviso finds no place in the prayer for "such favourable weather that we may in due time gather in the fruits of the earth," or for the prisoners that they may be brought "back to their homes in safety." Our petitions in these and other directions are not always granted. But why deliberately suggest that it may be God's will that the sick and wounded shall not be rescued from death?

What minister of Christ, from Archbishop to Deacon can retain his sacred office if he doubts in his heart that the wish of our Saviour is that the disabled shall be restored to health. Why should the Archbishops suggest that God's will may be that the sick and wounded shall not be rescued from death? We must have confidence in God's love for man or we are none of His. Hence the doctor and the nurse are right, and the doubters are wrong in the course they respectively adopt towards sickness and the wounded. Any army of doubting ministers, whatever else they may be, cannot be an army of Christ's representatives and pastors. We are in the throes of the most deadly of wars, which is awakening the apprehension of all classes to the needs of their spiritual nature. But "if the trumpet shall give an uncertain sound who shall prepare himself to the battle?"

Mr. Hayes Fisher, President of the Local Government Board, visited the Lord Mayor Treloar Cripples' Hospital and College at Alton on the 13th inst., and was very complimentary to the staff, as well he might be, seeing that 90 per cent. of these "incurables" are treated successfully. The system under which training for after independence goes hand in hand with medical and surgical treatment and skilled nursing has been amply justified by results. Besides setting a new current of hope stirring in these maimed lives, it has had its share in helping to shape some of the most successful schemes which have been framed to bring back health and independence to wounded soldiers. Mr. Hayes Fisher told his hearers there were 50,000 crippled children (the Treloar Home provides for 270), and that it was the nation's duty to alleviate the sad condition of all the others. He alluded to the passing of the Maternity Bill as a measure calculated to stop this terrible wastage at its source, and spoke with strong conviction of the responsibility resting on every individual to take part in the saving of child life. "We must not act merely as passengers in the boat. Each one of us must pull an oar." Such a united effort would lift the disgrace of the crippled child from the Mother Country within twenty years.

The fact that the administrative heads of the four largest training-schools for nurses in London had the honour of being selected for inclusion among the first recipients of the Order of the British Empire, is acclaimed in St. Bartholomew's

**ROUND THE HOSPITALS—*(continued)*.**

Hospital Report "as a further recognition of the invaluable services rendered in connection with the war by the nursing profession as a whole." Miss Cutler, assistant matron at St. Bartholomew's, has been awarded the Mons Ribbon and Star, 1914. Out of sixty staff nurses who passed out of the hospital in the year, twenty-five only, an unusually small proportion, joined the Naval or Military Services. The private nursing staff took twelve; and of the remainder, eighteen accepted sisters' posts in various other hospitals, four left to be married, and one to study medicine. Up to the present 132 nurses have been supplied from St. Bartholomew's for the Naval and Military Services, besides the twenty-four nurses of the T.F.N.S. called up in August 1914. So far as is known, no fewer than 346 Bartholomew's nurses are or have been doing war-work. A decrease to the extent of 500 was noticeable in the number of applications for the rules and regulations for probationers, but as the number even so amounted to over eleven hundred, no difficulty was experienced in selecting suitable candidates. Probationers' salaries have been raised from £8, £12, £20 to £14, £18, £22.

The Victorian Order of Nurses for Canada offers a post-graduate course in district nursing and social service work which takes four months, and may be taken at one of the training-homes of the Order—Toronto, Ottawa, Montreal, and Vancouver. The board of governors have recently been urged by the Victorian Order of Nurses to increase the efficiency of their four training centres and to open them to the pupils of all the standard training-schools of Canada in their last years of training. By this means the course will be taken as part of the regular training instead of being post-graduate, and if the training-schools avail themselves of it the supply of nurses will be speeded up. The difficulty about all these linking systems is to keep the wards going when nurses are withdrawn from them to continue their training on lines valuable to themselves and to the community, but outside the scope of their own training-school. In this country, at any rate, there is a vast deal of spade work to be accomplished before the wider needs of the community come to have full weight in shaping the course of the nurse's training.

Has anyone noticed the change which has insensibly crept over the use of the word "nurse" since the war began? "Nurse Smith" in common parlance, in both civil and military establishments, is either the untrained V.A.D. helper or the probationer—very rarely indeed a woman who has received her full certificate. The men are keen to note the distinction between the mere "nurse" and the "sister." In many establishments they salute the latter, while they expend merely a nod or friendly grin on the "nurses." We have noted in the *Times*, which is a recognised authority on nomenclature, that the expression "*nursing sisters*" is invariably used of trained nurses who come before the eye of the public, either by reason of danger incurred or honour bestowed, for the public does not understand the fine shade of distinction which in certain services and institutions prescribes a term as "staff nurse" before the full dignity of sister is reached. Would not the term "nursing sister" be a more gracious and recognisable description for trained nurses when registered by the State than merely "registered nurse"? If none but nurses who had been accepted by the State as fully trained were permitted to describe themselves as "nursing sisters" it might safely be prophesied that the proposed voluntary system of State Registration would speedily become universal. No trained nurse could afford to forgo her right to this title, which has its roots in mediæval days when women first studied the science and art of nursing.

The suggestion of short popular leaflets setting forth the moral and material attractions of the nurse's life is an excellent one. We have some very able writers and leaders amongst the members of the nursing profession, both here and in the United States, as well as an army of hospital leaders, many of whom have considerable literary gifts and also full knowledge. These, we are sure, will gladly co-operate in the preparation of such leaflets for the good of the profession, but we wish to give all able to produce such leaflets a little encouragement, and have therefore decided that if not less than thirty competitors send their names to the Editor by Saturday, August 17, and thirty sets of leaflets are accepted as good by the judges appointed, we will give three prizes, viz. three guineas for the best, two guineas for the second best, and one guinea for the third set of leaflets so classed by the judges. All intending competitors should send in their names and addresses to the Editor of The Hospital, and all leaflets for competition must reach him at 28 Southampton Street, Strand, London, W.C. 2, not later than Saturday, September 28, 1918.

The news of the coal ration has struck the nation with dismay. Institutions will feel the pinch even more severely, it may be feared, than private houses, for among numbers it is far more difficult to control supplies. A roaring fire half way up the chimney has been the accepted note of cheerfulness in every ward during black weather, and to say its size must now be reduced by half is to understate the case. The first essential to economy is to reckon out the winter amount allowed per day according to the ration for the whole establishment. The next is to weigh the contents of every coal receptacle used in the institution and place over each card stating the number of pounds it holds and the amount which can be allowed under the ration to that particular room. That much economy in fuel can be effected without inflicting discomfort on any one hardly admits of a doubt. But economy will have to be carried far beyond this point to meet the coal ration.

**ROUND THE HOSPITALS—(*continued*).**

THERE is reason to fear that the salaries of sisters and matrons are hardly keeping pace with the upward tendency observable in those of probationer nurses. In our issue of July 20 we had the pleasure of announcing that the Taunton and Somerset Hospital had made additions to their probationers' pay of £7, £8, £9, and £5 in their respective years of service, but we have had no information that the sisters have received an increase. It is still the custom to regard sisters' posts as part of the training in the sense that it increases the value of the nurse to hold them. It is true in all good training-centres that nurses are always ready to take such work on for a year or two at a very moderate remuneration before passing to a fresh sphere, and that there is usually no lack of applicants for sisters' posts. This, of course, tends to keep down salaries. Some matrons consider it a disadvantage to the training-school that sisters should be induced to stay on at their posts indefinitely. They prefer a succession of younger women, keen and fairly recently trained, professedly holding office as a preliminary to other work. These considerations have not hindered the authorities in some wisely administered hospitals from the act of justice involved in raising the pay of the sisters on whom the prosperity of their training-schools may be said to depend. The fact that sisters enlarge their experience in the course of performing their duties ought not to be made a reason for underpaying them.

---

THE Colonial Nursing Association has moved with the times in altering its title to the Overseas Nursing Association. Its scope has been widely extended since it was founded, and the term "Colonial" is now no longer applicable. Truth to tell, the word "Colonial" is not in particular favour in the Empire. It savours of the backwood and of a dependence on the initiative of the Mother Country now generally undesired. In some of the places formerly supplied with nurses from Home they are now happily training their own nurses, and have flourishing training-schools. But there is now equal, and will soon be greater, scope for the activities of the Overseas Nursing Association and need for its further expansion, as is illustrated by the recent despatch of two nurses to German East Africa. The sisters attached to the Service tend to remain at their posts for a long term of years, and it is a fine record that nineteen silver badges for meritorious service for five years and upwards have been awarded. No branch of nursing work has been more hampered by the war than this. Two sisters, Miss Graham and Miss Poulter, lost their lives through submarine attacks on the vessels *Abasso* and *Appapa* last year.

---

QUEEN VICTORIA'S JUBILEE INSTITUTE FOR NURSES, with 592 of its nurses now undertaking duty in connection with the war, has a hard struggle to keep all its districts served. The tale of its doings, shown in the recently issued report for 1917, bears witness to indomitable courage displayed by nurses, superintendents, and inspectors in carrying on. The demands for their services are increasing. The supply of new recruits to the Institute is shrinking. All along the line it is a record of hard work accomplished by a faithful and too small band of workers. The number of fresh Associations affiliated in the past year was 28, making a total of 1,034. The County Nursing Associations in affiliation now number 34, and the Queen's Nurses on January 1, 1918, numbered 2,006, including those on war duty. With village nurses, midwives, and probationers in training the total in the United Kingdom was 4,376. Only 105 nurses were placed on the Roll of Queen's Nurses last year, as compared with 144 the year before, but the Council may be congratulated that, in spite of all the insistent claims on women in general, and nurses in particular, they have won through without loss of efficiency or any lowering of the standard.

---

IN matters of internal administration the Council of the Jubilee Institute has made progress of an important nature. For the first time in its history the Queen's Nurses have direct representation on the governing body, the superintendents of the training-homes and the superintendents of the County Nursing Associations being each allowed to elect a representative. Moreover, as some of the representatives of the affiliated Associations find it difficult to attend meetings of the Council regularly, on account of their distance from London, a system of alternative or substitute members has been devised. The representatives who live 100 miles or upwards from London are allowed, with the approval of the Association which has elected them, to propose another member for nomination who, in their absence, may attend the meetings of the Council with full powers, including that of voting. Three alternative members have already been appointed under this scheme, which, should it prove successful, will probably meet with imitation. It would greatly strengthen many Councils which depend for their influence on the adhesion of members from all parts of the country if the appointment of alternative members were more generally admissible.

---

MORE than 500 College women have already registered for the special course in preparation for nurses' training-schools offered at Vassar College this summer. The Chief Inspecting Nurse of the War Department, in concert with the highest medical and nursing authorities in the United States, has under consideration a plan for an Army School of Nursing, whereby nurse-training schools will be established in cantonment hospitals. The idea is to minimise the volunteer element and replace it wherever possible by women specially trained for the particular branch of military nursing, even if not qualified to undertake general work. The American experience of the amateur or volunteer nurse does not appear, so far, to have been as happy as our own.

**For the Making of Profit and the British Nurse, by "Ierne," see p. 370; The London Hospital Nurses: A Race between Statesmanship and Economics, 375; Matrons' and Sisters' Appointments, see p. 378.**

